# Structural and Dynamic Analyses of Pathogenic Variants in *PIK3R1* Reveal a Shared Mechanism Associated among Cancer, Undergrowth, and Overgrowth Syndromes

**DOI:** 10.3390/life14030297

**Published:** 2024-02-23

**Authors:** Nikita R. Dsouza, Catherine E. Cottrell, Olivia M. T. Davies, Megha M. Tollefson, Ilona J. Frieden, Donald Basel, Raul Urrutia, Beth A. Drolet, Michael T. Zimmermann

**Affiliations:** 1 Computational Structural Genomics Unit, Genomics Sciences and Precision Medicine Center, Medical College of Wisconsin, Milwaukee, WI 53226, USA; 2 The Steve and Cindy Rasmussen Institute for Genomic Medicine, Nationwide Children’s Hospital, Columbus, OH 43205, USA; 3 Department of Pathology, The Ohio State University, Columbus, OH 43210, USA; 4 School of Medicine, Medical College of Wisconsin, Milwaukee, WI 53227, USA; 5 Department of Dermatology and Pediatrics, Mayo Clinic, Rochester, MN 55905, USA; 6 Department of Dermatology, University of California-San Francisco, San Francisco, CA 94143, USA; 7 Division of Genetics, Department of Pediatrics, Medical College of Wisconsin, Milwaukee, WI 53226, USA; 8 Department of Surgery, Medical College of Wisconsin, Milwaukee, WI 53226, USA; 9 Department of Biochemistry, Medical College of Wisconsin, Milwaukee, WI 53226, USA; 10 Department of Dermatology Pediatrics, School of Medicine and Public Health, University of Wisconsin, Madison, WI 53715, USA; bdrolet@dermatology.wisc.edu; 11 Clinical and Translational Sciences Institute, Medical College of Wisconsin, Milwaukee, WI 53226, USA

**Keywords:** precision medicine, genomics, genomic data interpretation, overgrowth, undergrowth, PI3K, PROS

## Abstract

The PI3K enzymes modify phospholipids to regulate cell growth and differentiation. Somatic variants in PI3K are recurrent in cancer and drive a proliferative phenotype. Somatic mosaicism of *PIK3R1* and *PIK3CA* are associated with vascular anomalies and overgrowth syndromes. Germline *PIK3R1* variants are associated with varying phenotypes, including immunodeficiency or facial dysmorphism with growth delay, lipoatrophy, and insulin resistance associated with SHORT syndrome. There has been limited study of the molecular mechanism to unify our understanding of how variants in *PIK3R1* drive both undergrowth and overgrowth phenotypes. Thus, we compiled genomic variants from cancer and rare vascular anomalies and sought to interpret their effects using an unbiased physics-based simulation approach for the protein complex. We applied molecular dynamics simulations to mechanistically understand how genetic variants affect PIK3R1 and its interactions with PIK3CA. Notably, iSH2 genetic variants associated with undergrowth destabilize molecular interactions with the PIK3CA receptor binding domain in simulations, which is expected to decrease activity. On the other hand, overgrowth and cancer variants lead to loss of inhibitory interactions in simulations, which is expected to increase activity. We find that all disease variants display dysfunctions on either structural characteristics or intermolecular interaction energy. Thus, this comprehensive characterization of novel mosaic somatic variants associated with two opposing phenotypes has mechanistic importance and biomedical relevance and may aid in future therapeutic developments.

## 1. Introduction

As genetic sequencing of individuals becomes an increasingly common tool for precision medicine, we are discovering that specific genes that orchestrate fundamental physiologic functions can be responsible for phenotypes with opposing effects. This emerging evidence is critical to examine as it differs from a historical view that each gene produces a single disease when altered. We aim to broaden our understanding of such pleiotropic effects when investigating disease-associated genomic variation in the gene encoding phosphatidylinositol 3-kinase receptor (PIK3R1), which binds a catalytic subunit (PIK3CA) to make the heterodimeric functional enzyme (PI3K). Germline variants in *PIK3R1* can be associated with autosomal dominant growth delay and insulin resistance (SHORT syndrome: short stature, hyperextensibility of joints/hernia, ocular depression, Rieger anomaly, and teething delay) [1,2,3] and are also described in the setting of immunodeficiency (Activated PI3K-Delta Syndrome 2, APDS2) [4]. Somatic alteration in *PIK3R1* comprises an overlapping variant spectrum between vascular malformation and overgrowth to that of cancer in association with the dysregulation of the PI3K enzyme [5,6,7,8,9]. Thus, PI3K exhibits opposing phenotypes [10] across its observed mutations and is a medically relevant testbed for bringing new approaches beyond genomics to enhance the mechanistic interpretation of human genetic variation.

PI3K enzymes function by phosphorylating phospholipid head groups and marking proteins associated with the cell membrane. The complex formed by PIK3CA and PIK3R1 is critical for modifications that regulate AKT signaling and, thereby, cellular metabolism, cell cycle, and apoptosis. Both are multi-domain proteins, each domain of which has distinct but interrelated functions. Briefly, PIK3R1 has two domains that directly interact with PIK3CA—first, a Src Homology 2 (SH2) domain. In general, SH2 domains bind to other proteins at phosphotyrosine residues. The interaction between PIK3R1 and PIK3CA is an exception to this rule [11]. The interaction between the N-terminal SH2 (nSH2) domain of PIK3R1 (defined by CATH as spanning amino acids 317–430) and PIK3CA is inhibitory by competition assays using other naturally occurring phosphorylated protein targets [12], phosphotyrosine peptides [13], and genetic studies [14,15]. In addition, PIK3R1 contains an “inter-SH2” (iSH2, amino acids 440–600) domain, so named because it is flanked by SH2 domains in the protein sequence, for which the previously mentioned studies identify a scaffolding and stabilizing role. However, a 3D molecular approach is needed to clarify the mechanism for why the PIK3CA-PIK3R1 interaction is an exception to the classic SH2-binding rules. Moreover, this knowledge has implications for discovering novel molecular mechanisms underlying the pathogenic function of cancer hotspot mutations and the genomic variants that drive undergrowth and overgrowth syndromes.

Given the pleiotropic effects of *PIK3R1* variation, it becomes vital to understand the underlying molecular mechanisms. Even highly studied genes, such as those comprising PI3K, frequently exhibit variants of uncertain significance (VUS), which precludes their interpretation for diagnosis and mechanistic understanding. Interpreting VUS identified through next-generation sequencing typically starts with annotation, which considers overlapping or closely associated sequence-based features [16,17]. In the case of *PIK3CA*, observation of the same variant across similarly affected individuals has allowed for clinical diagnosis [18]. Still, it has not extended our understanding of the functional mechanism underlying variant pathogenicity. Standard annotations used to understand the impact of a genomic variant are its potential for pathogenicity as determined by the frequency at which it is found in the population, the frequency observed in disease, and the predicted change in the protein-coding sequence. However, biological mechanisms are carried out by the gene product, a protein with a particular 3D structure and movements [19,20,21]. Limited information is gleaned from sequence-based predictions of pathogenicity, which are primarily based on sequence conservation and are frequently limited to missense variation. Sequence-based prediction typically does not inform how the 3D molecule is altered since atomic properties, such as bonds among proteins, cannot be determined using such an approach. Thus, combining sequence-based annotations, 3D molecular modeling, and knowledge of cell biology will enable us to develop more integrated functional models to interpret VUS and deconvolute their distinct pathobiological roles mechanistically. Therefore, in this study, we applied molecular modeling, molecular mechanic calculations, and dynamics simulations to understand better how genetic variants observed in diverse phenotypes affect PIK3R1 and its interactions with PIK3CA at an atomic resolution level. We specifically chose representative variations from undergrowth and overgrowth syndromes, cancer, and a healthy population reference, to inform this knowledge gap. The results of our study reveal that the loss of inhibitory SH2 interactions drives cellular growth, while the loss of activating iSH2 interactions restricts cellular growth. These predictions from our modeling integrate data from germline, somatic mosaic, and cancer studies into one mechanism, supporting their pathobiological relevance and providing a framework for interpreting novel variants identified in future studies. Therefore, these data bear significant relevance to the field of precision medicine by contributing to both rare and undiagnosed diseases and cancer caused by genomic variation in *PIK3CA* and *PIK3R1.*

## 2. Materials and Methods

### 2.1. Selecting and Annotating PIK3R1 Genomic Variants

Genetic alterations to PI3K, including the receptor PIK3R1, are known to have pleiotropic effects spanning cancer and heritable syndromes that have components of under- and overgrowth, among other features (Table 1). The mechanism of how changes to the same protein produce phenotypes with varying directions of effect is highly interesting. For the current study, genetic variants were identified as missense and indel variants with low allele fraction, as previously described [6,22], and from cancer specimens [23]; individual consent was not needed in the current study since we are studying the difference that genomic variants, selected from previous research, impart on protein models. The cohort comprised 17 patients with PIK3R1 variants from 3 institutions [6]. Most PIK3R1 variants were observed as mosaic, typically under 10% allele fraction. Somatic mosaic variants overlapped with the regions of known cancer hotspots [24,25]. We also selected two iSH2 genetic variants for the SHORT syndrome: E487S [26] and F487S [27] (Table 2).

### 2.2. Molecular Modeling and Molecular Dynamics Simulation of PI3K

**Figure 1:**
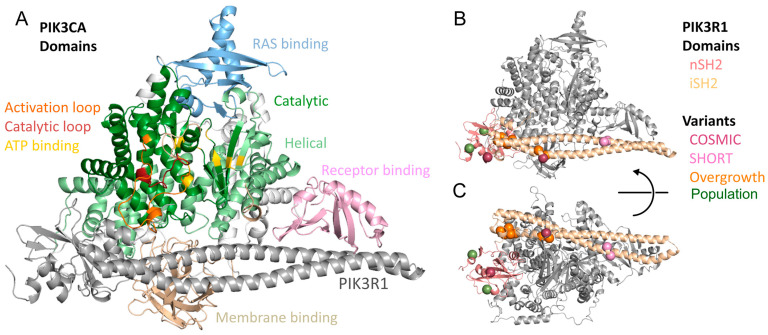
Overview of PIK3R1 and PIK3CA structure and location of studied variants. (**A**) This study focuses on variants observed in PIK3R1; the effects of the variants must be interpreted in terms of their effects on the protein complex and its enzymatic function. We here depict the pro PIK3R1 and PIK3CA protein complex and annotate domains and functional sites of PIK3CA with PIK3R1 colored gray. (**B**) We show the complex with PIK3CA now colored gray and two domains of PIK3R1 within our structural model individually colored. Sites of studied variants (large spheres) are colored according to their associated phenotype. (**C**) A rotated view better shows the difference in orientation and PIK3CA interactions for the two PIK3R1 domains.

We used molecular modeling to generate a model of the PIK3CA:PIK3R1 interaction using customized inputs to Modeller [31] 9v8 and with individual components determined by advanced homology-based methods [32]. Molecular modeling leveraged multiple experimental structures, primarily the human wildtype (WT) complex solved with PIK3CD (Protein Data Bank, PDB, ID: 5itd [33]) or mTOR inhibitor (PDB: 2l2y [34]). We generated structural models of missense variants using FoldX v4.0 [35,36] and in-frame insertion–deletion (indel) variants using interactive molecular mechanics followed by energy minimization [37]. Specifically, from our WT model, we deleted the affected amino acids that are aberrantly spliced out, performed two rounds of molecular mechanics geometric optimization on the two amino acids on each side of the event, followed by ten rounds of molecular mechanics geometric optimization on the four amino acids on each side of the event, and finally energy minimization of the region within 12 Å around the event. This procedure produced our initial indel models. We used Pfam [38] and CATH domain classification to visualize PIK3R1 and highlight the domains on our 3D model, showing the N-terminal SH2 domain (nSH2) and the inter-SH2 (iSH2) domain, which includes two long helices that wind around one another (Figure 1). For the Molecular Dynamics (MD), we computed generalized Born implicit solvent MD simulations using NAMD [39] and the CHARMM27 with CMAP [40] force field. We used an interaction cutoff of 12 Å with strength tapering (switching) beginning at 10 Å, a simulation time step of 1 fs, and conformations recorded every 2 ps. To simplify the degrees of freedom in the system, the motion of PIK3CA was constrained using internal quadratic harmonic constraints. We used each initial conformation to generate three replicates, and each was energy minimized for 5000 steps, followed by heating to 300 K over 300 ps via a Langevin thermostat. We generated a further 15 ns of simulation trajectory for each replicate, and the final 10 ns were analyzed. Using the same procedure, we ran another set of independent triplicate simulations at 360 K. Thus, we generated nearly 100 ns of MD trajectory for each variant. We aligned all trajectories to the initial wild-type conformation using Cα atoms of PIK3CA and the CE algorithm [41]. Trajectories were analyzed using custom scripts, leveraging VMD [42] and the Bio3D R package [43]. Protein structure visualization was performed in PyMol v1.9.0 [44].

### 2.3. Statistical Analysis

We calculated Root Mean Squared Deviation (RMSD) and Cartesian space principal component (PC) analysis using Cα atoms of PIK3R1. We chose specific Cα atoms as markers for distance monitors between iSH2 helices: PIK3R1 p.K567 to PIK3CA p.E453, PIK3R1 p.R577 to p.Y452, and PIK3R1 p.V445 to p.L584. These distance monitors were used to assess the level of local unfolding at the end of the helical domain. We generated Free Energy Landscapes (FELs) of the motions apparent within PCs using Karamzadeh et al.’s approach [45], which is based on the time-dependent joint probability between each PC motion.

MD trajectory data have a dense time resolution, yielding many observations for each simulation. We used a down-sampling procedure for a more conservative calculation of statistical significance. We randomly and repeatedly selected 100 conformations from each trajectory, calculated each measurement (e.g., interaction energy), and compared between variants using a *t*-test. We used the median t-statistic from 1000 repetitions to assess statistical significance. This procedure was used for distance measures across MD trajectories and MD-based PC differences, in comparison to WT.

### 2.4. SH2 Domain Representatives

We queried the PDB [46] for human SH2 protein complexes, defined by membership in the Pfam [38] family PF00017. We required that the structures chosen contain a phosphotyrosine residue, defined at a 1.5 Å crystallographic resolution or better, and less than 90% mutual sequence homology. These criteria identified eight representative structures with PDB IDs: 5gjh, 5gji, 5aul, 4u1p, 3wa4, 2vif, 2cia, and 1lkk. We selected the first biologic unit of each representative structure for comparison to the SH2 domain of PIK3R1. We superimposed them onto the PIK3R1 SH2 domain using the CE (combinatorial extension) algorithm [41] as implemented in PyMOL.

## 3. Results

### 3.1. Structure-Based Assessment of PIK3R1 Variants

To mechanistically characterize the pleiotropic effects of PIK3R1 genetic variation (Table 1), we first mapped variants observed in germline and somatic disease throughout the 3D structure of PIK3R1. All were near multiple domains of PIK3CA (Figure 1, Table 2). Two alleles that cause the undergrowth phenotype SHORT are within the PIK3R1 iSH2 domain and across from the PIK3CA receptor-binding domain. Five overgrowth case variants are spread throughout the middle of the iSH2 domain and are not at the protein interface to PIK3CA. The same five, plus an additional nSH2 variant, R409Q, are also cancer variants observed in human tumors. We also modeled the effects of two distinct in-frame deletions on structure-based features. Like our study of missense variation, we aimed to understand the details underlying how INDELs associated with overgrowth syndromes may affect the stability and dynamics of PIK3R1 (Figure S1). Further, we tested the hypothesis that missense and deletion alleles share a common molecular mechanism. In the sections below, we considered how missense and deletion alterations affect the structure and dynamics of the receptor and, thereby, interaction with PIK3CA.

### 3.2. Mechanism of Somatic Hotspot Variants

**Figure 2:**
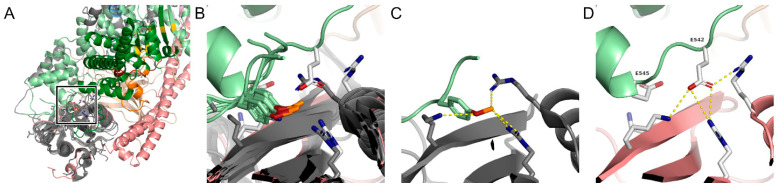
Cancer hotspot residues determine SH2 binding specificity. (**A**) Eight independent high-resolution experimental structures of different protein’s SH2 domains bound to phosphopeptides are colored dark gray and superimposed onto the PIK3R1 SH2 domain; other domains are colored as in Figure 1. The bound peptides are colored light green. (**B**) We zoomed in on the boxed region from (**A**) to show the consistency of the binding interface. Sticks are shown for the phosphotyrosine and interacting side chains from PIK2R1 and one representative example—GRAP2 SH2 domain bound to CD28-derived phosphopeptide (PDB 5GJH). (**C**) GRAP2 is shown individually and the charge interactions with the phosphotyrosine residue shown as yellow dashed lines. (**D**) We similarly show PIK3R1. Cancer hotspot variant sites are analogous to the classic phosphotyrosine of other SH2 domain targets. Thus, the phosphomimetic aspartic acid residues explain the phosphorylation-independent SH2 binding of PIK3CA to PIK3R1.

Since the activation of PI3K through cancer hotspot variants is well established, we first investigated their structural and dynamic molecular properties that can inform their mechanism. For this purpose, we compared distinct biophysical details that describe the interactions between PIK3CA and PIK3R1 using eight high-resolution experimental structures of human SH2 domains bound to phosphotyrosine peptides derived from their natural targets (Figure 2). This comparison confirmed that the PIK3CA cancer hotspot sites, glutamic acid residues, p.E542 and p.E545, naturally mimic the phosphotyrosine residues that most SH2 domains require for specific binding. This highly conserved structural feature of SH2 domains allows interpretation of the molecular mechanism for why the cancer hotspot variants (p.E542K and p.E545K [5]) are seen with high recurrence—they are precisely the residues that determine specificity between the receptor SH2 domain and PIK3CA. Because SH2 domain binding is inhibitory [13,14], we expect a loss of specificity, without any loss of affinity along the reset of the PIK3R1:iSH2 interface, to activate PIK3CA. Therefore, alteration to the PIK3R1 SH2 domain or its interactions with PIK3CA would be consistent with the effects of the cancer hotspot mutations of PIK3CA.

### 3.3. Mechanism of Germline Syndromic Variants

**Figure 3:**
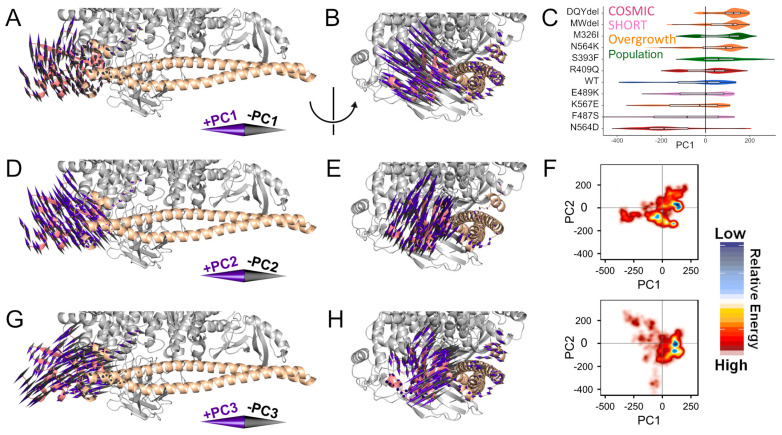
PIK3R1 variants alter nSH2 domain positioning. We summarized the motions apparent within our MD simulations using PCA. Each PC vector indicates a motion of the protein complex. We visualized the first motions using a cone for each residue, focusing on the residues with the greatest motion. Coloring is as in Figure 1B. Animations of these motions are available in our Supplemental Data. (**A**,**B**) We show the first PC (PC1) motion which corresponds to the movement of the PIK3R1 SH2 domain towards (+PC1) or away (−PC1) from PIK2CA. (**C**) PC1 can be used as a score to indicate how the motion is differentially activated for each variant. (**D**,**E**) We show PC2 motion which corresponds to the movement of the PIK3R1 SH2 domain orthogonal to PC1. (**F**) Multiple PC motions are combined to generate a free energy landscape (FEL). The FEL for WT and the polymorphism M326I (upper) is distinct from that of the VUS observed in association with overgrowth syndromes (lower). Additional FEL images are available in Figure S3. (**G**,**H**) We show PC3 motion which corresponds to the movement of the PIK3R1 SH2 domain, reminiscent of PC2 but the domain no longer moves as a complete domain. Rather, two halves of the SH2 domain spread apart as they move towards the catalytic center (+PC3), or fold together as they move away (−PC3).

We seek to distinguish the molecular mechanisms of SHORT variants from those of overgrowth variants in PIK3R1. To this aim, we used MD simulations to study the time-dependent effects of each variant in high resolution. The details of how each variant affects the structure or dynamics of PIK3R1 may differ, but we are interested in the overall effects. Thus, we summarized the motions apparent within our MD simulations using PC analysis (Figure 3). Each PC vector indicates a large-scale movement of the protein complex; animations of these motions are available in our Supplemental Data. The dominant movements are changes to the orientation or binding of the PIK3CA SH2 domain at the PIK3CA interface. Thus, we were able to observe un-docking events in simulations, highly supportive that iSH2 alteration can regulate SH2 inhibitory binding by allostery and supporting our hypothesis that overgrowth variants phenocopy somatic hotspot functions.

### 3.4. Vascular Anomalies and Overgrowth

**Figure 4:**
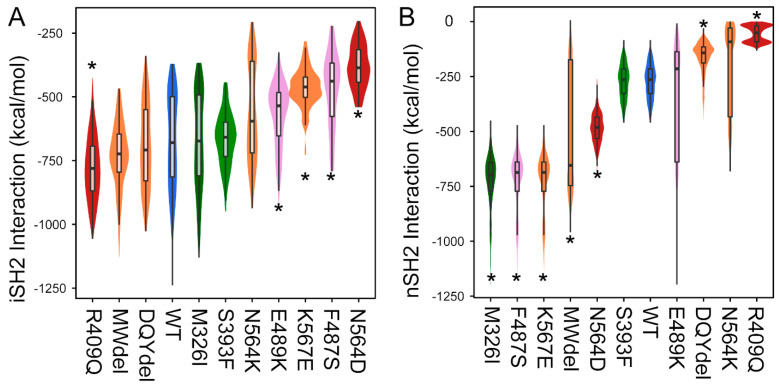
Variants alter interaction energy for specific domains. We summarize interaction strengths using smoothed kernel density (violin) plots with boxplots overlaid and coloring as in Figure 1. An asterisk indicates statistical significance (*p* < 1 × 10^−6^). (**A**) First considering the iSH2 domain of PIK3R1, four variants (E489K, K567E, F487S, and N564D) significantly diminished the interaction strength with PIK3CA. Note that the interaction surface between the two proteins is extensive, making the interaction strength reflective of many individual interactions. (**B**) Similarly, for the first SH2 domain, three variants were associated with significant destabilization (R409Q, N564K, and DQYdel), while five with significant stabilization. Because the SH2 domain binding is inhibitory, we expect increased interaction to be associated with decreased activity.

We have identified somatic mosaic variants in PIK3R1 harbored within the disease-affected tissue of individuals with vascular anomalies and overgrowth syndromes. In MD simulations, these variants convey consistent changes to the structure of PIK3R1. The genetic variants p.R409Q (described in cancer) and p.N564D (described in cancer and vascular/overgrowth) had interaction energy that resulted in the former destabilizing the binding to PIK3CA and the latter stabilizing the binding significantly (Figure 4). This is also supported by the PC analysis that shows the R409Q model moves PIK3R1 away from PIK3CA with a median PC1 shift of 0.31 and N564D moves more towards PIK3CA with a median shift of −1.84 compared to the WT (Figure 3). We observed a consistent shift towards a conformation where the SH2 domain projects further against PIK3R1, between the catalytic and iSH2 domains, and away from the membrane. In contrast, the SH2 domain itself moderately contorts such that the N- and C-terminal sections of the SH2 domain move apart from one another (Figure 3 and Figure S3). The overgrowth variants MWdel (p.(Met582_Asp605delinsIle; Exon 14 skipping), DQYdel (p.(Gln579_Tyr580del) and p.N564K shift PC1 such that the SH2 domain moves away from PIK3CA, the SH2 domain of p.K567E moves the SH2 domain closer to PIK3CA with a stabilizing SH2 interaction energy, possibly keeping it bound to PIK3CA. MWdel also stabilizes SH2 interaction energy, whereas DQYdel and p.N564K have a significantly destabilizing interaction energy affecting the PIK3R1:PIK3CA binding (Figure 3 and Figure 4). The distance between the PIK3CA and the iSH2 domain also increases (Figure S2). Because the interaction between PIK3CA and the SH2 domain of PIK3R1 is inhibitory, we expect a loss of this interaction to increase activity, in agreement with an overgrowth phenotype.

### 3.5. SHORT Syndrome and Undergrowth

Pathogenic variants for SHORT syndrome are enriched within the cSH2 domain of PIK3R1 and observed in the iSH2 domain. These variants destabilize the interaction with the PIK3CA RBD domain (Figure 4). The interaction between PIK3R1:iSH2 domain and PIK3CA:RBD is a known activation mechanism. Thus, we expect the loss or destabilization of this interaction to decrease activity in agreement with an undergrowth phenotype. Based on the interaction energy calculation for the helical and RBD binding domain, we see that both the short variants p.F487S and p.E489K are significantly destabilizing (Figure 4). The interaction energy calculations show that this destabilization could decrease activity and the resulting undergrowth phenotype.

## 4. Discussion

The current manuscript extends our understanding of disease-associated genomic variations in the PIK3CA gene in areas that relate to disease mechanisms and likely future diagnostics and therapeutics. Indeed, using highly parametric, computational biophysical methods to extend previously derived experimental data, we have developed a high-resolution structural and dynamic molecular model that explains functional alterations in the PI3K/PIK3CA complex associated with diseases caused by germline and somatic genetic alterations. A premise of this model is that the proper enzymatic activity and biological function of PI3K is driven by a balance between receptor binding affinity, mainly through the iSH2 domain, inhibitory docking of the receptor SH2 domain, and specificity of the SH2 domain for its target. Therefore, these factors, influence the orientation of the catalytic domain concerning the membrane, thereby modifying accessibility to the active site.

Our model clarifies the molecular mechanisms underlying opposing phenotypes affecting the same gene, which is challenging to predict using statistical methods, and provides insight into how similar approaches could be used for other gene products. In this way, the current study highlights the utility of molecular modeling for clinical genetics and precision oncology. We compared structural features of other SH2 interactions to determine that the PIK3R1 somatic hotspot sites appear to phosphomimic the classic phosphotyrosine that gives SH2 regulatory specificity in other enzymes. Using this knowledge, we used physics-based Molecular Dynamics simulations to understand the effect of genetic variants on complex stability. From simulations, vascular anomalies, and overgrowth variants in the iSH2 domain were observed to cause the latter to un-dock from PIK3CA, mimicking the expected somatic hotspot behavior. The interface configuration for the cancer hotspot residues differs from the classic phosphotyrosine-binding configuration. We believe this difference allows for competitive binding of other PIK3R1 target proteins. The cancer hotspots lose their phosphomimetic nature with a charge reversal, negating the binding of PIK3CA and PIK3R1 through this domain. However, binding through the iSH2 domain may be independent of the SH2 domain. As the field of precision oncology continues to develop and test inhibitors of the PI3K/AKT pathway [47], our results demonstrate that companion testing approaches will likely be needed to understand better how different mutations may activate or inactivate the enzyme, leading to increased or decreased efficacy when pharmacologically inhibited. Additional information will be gained when integrated with proteomics and artificial intelligence systems [48]. Thus, the current study bears significance for PI3K disorders and precision oncology.

Another important contribution of this study is its application to the mechanistic interpretation of genomic variants in other PI3K family members like PIK3CD. PIK3CD, like PIK3R1, has a molecular mechanism similar to APDS2 [49]. Therefore, the vascular malformation/overgrowth variants in the iSH2 domain affect the binding of the iSH2 domain with the PIK3CA, resulting in the overgrowth phenotype. The SHORT variants in this study affect the binding between the helical domain and RBD, resulting in the undergrowth phenotype. We support this interpretation using molecular simulations and computational characterization of the interactions within the protein complex. Protein complex interactions occur dynamically in 3D through non-linear features and are modifiable by allosteric communication. The model that we have developed and used herein captures one state (arrangement of the receptor and enzyme) and context (folded and membrane-bound). While the gain in mechanistic insights is evident, this model does not account for potential changes to gene expression levels, mRNA structure, the protein folding process, or other states and contexts where PI3K may act. Thus, approaches such as the ones we have applied here are required for translational genomics to understand the implications of novel human genetic variation in high mechanistic resolution.

In summary, our study uses well-established basic science tools of physics-based protein simulations to calculate the effects of PIK3R1 disease-associated alleles on the enzymatic complex, which added detail to our understanding of how genomic variants affect PI3K function. Because selected variants used in this study have been previously assessed experimentally, they act as benchmarks for comparing variants that have not been experimentally evaluated. Our future work will expand the number of variants assessed using computational and in vitro approaches, including those observed in PIK3CA-associated diseases, rarely in the currently health population [50] and for other PI3K family members. Additional molecular models that account for further states and contexts where PI3K acts will illuminate the additional details of its physiologic and pathobiological roles across cancers and developmental disorders. We are optimistic that this type of investigation will add significant interpretative value to the growing data derived from the increased application of NGS to medical research and diagnostics.

## 5. Conclusions

PIK3R1 variants play a significant role in vascular malformation and overgrowth for our cohort. In this study, we propose a functional and mechanistic interpretation of how somatic and germline variants affect the activity of the PI3K enzyme formed by PIK3CA:PIK3R1. The loss of affinity at the inhibitory PIK3R1-SH2 interface can drive cellular growth, while the loss of affinity at the activating PIK3R1-iSH2 interface restricts cellular growth. Specifically, SHORT variants destabilized (F487S and E489K) interactions with the iSH2 domain. In contrast, cancer and overgrowth-associated variants stabilized (R409Q) or destabilized (N564D and K567E) iSH2 interactions, while also stabilized (K567E, N564D, and MWdel) or destabilized (N564K, R409Q, and DQYdel) SH2 interactions. Thus, our study supports a common molecular mechanism underlying germline mosaic and cancer variants where either side of the inhibitory molecular interface is altered. Methods with higher resolution for functional interpretation of genomic variants, such as those described here, are critical for improving the diagnostic yield of clinical genomic sequencing and identifying opportunities to modulate the effects of functional genetic variants by identifying the most likely underlying molecular mechanism.

## Figures and Tables

**Table 1:** Pleotropic effects of PIK3R1 genetic variation.

	Cancer	Somatic Overgrowth/Vascular Malformation	SHORT Syndrome	Activated PI3K-Delta Syndrome 2
**Disease etiology**	Somatic variation in tumor	Post-zygotic somatic mosaic variation in affected cell lineages	Germline	Germline
**Reported Variation**	Missense, in-frame indel, splice [28]	Missense, in-frame indel, in-frame splice (exon 14) NM_181523	Nonsense, frameshift, missense, in-frame indel [29]	Missense, in-frame splice (exon 11) NM_181523
**Impact on PI3K complex**	Dominant, activating	Dominant, activating	Loss of function	Dominant, activating
**PIK3R1 Domain**	nSH2 and iSH2 enhanced	iSH2	cSH2 predominantly	iSH2

**Table 2:** Annotation of PIK3R1 variants analyzed in this study.

Variant	Label	Phenotype	MAF	Domain	ΔΔGfold	ΔiSH2 Interaction ^‡^	ΔSH2 Interaction	ΔPCs ^†^	ΔSH2	RMSD	CADD	SIFT	PPH2
p.M326I	M326I	gnomAD	1.8 × 10^−1^	nSH2	0.41	n.s.	+	+PC2, −PC3	Mod	-	17.5	T	B
p.S393F	S393F	gnomAD	5.9 × 10^−4^	nSH2	0.93	n.s.	n.s.	+PC2	High	High	33.0	D	D
p.R409Q	R409Q	Cancer *	8.0 × 10^−6^	nSH2	0.11	+	−	−PC3	Mod	Low	28.2	T	B
p.F487S	F487S	SHORT	-	iSH2	2.82	−	+		Mod	High	32.0	D	D
p.E489K	E489K	SHORT	-	iSH2	−0.18	−	n.s.	−PC3	Mod	-	26.7	D	D
p.N564D	N564D	Cancer/Overgrowth	-	iSH2	0.49	−	+	−PC1, +PC2, −PC3	High	High	28.3	D	B
p.N564K	N564K ^§^	Cancer/Overgrowth	-	iSH2	0.49	n.s.	n.s.	−PC3	Mod	Low	28.1	D	D
p.K567E	K567E	Cancer/Overgrowth ^	-	iSH2	−0.32	−	+	−PC3	High	-	30.0	D	D
p.(Gln579_Tyr580del)	DQYdel	Cancer Overgrowth	-	iSH2	n.s.	n.s.	−	+PC1, −PC3	High	Low	NA	NA	NA
p.(Met582_Asp605delinsIle); Exon 14 skipping)	MWdel	Cancer/Overgrowth	-	iSH2	n.s.	n.s.	+	+PC1	High	Low	NA	NA	NA

n.s., not significant. NA, not applicable. * This variant is also associated with altered insulin levels. ^ This variant is also observed in cancer as reported by COSMIC or occurs within a known hotspot [24,28]. ^†^ We indicate PC alteration if the variant’s median differs from the WT median by ≥1σ. ^‡^ As measured by interaction energy with “+” indicating stronger interaction and “−” indicating weaker interaction. ^§^ This patient exhibited clinical features of both MCAP and Activated PI3K-Delta Syndrome 2 (APDS2) [6,30].

## Data Availability

Subsets of 20 frames from each replicate of each of our simulations are stored in two data files: cached_subset_alpha_carbon_positions.RData, and cached_subset_alpha_carbon_positions_360K.RData. These data were used to score the dynamic effects of PIK3R1 genomic variants on the encoded protein and are included in Supplementary Materials.

## Supplementary Materials

The following supporting information can be downloaded at: https://www.mdpi.com/article/10.3390/life14030297/s1, Figure S1: WT 3D context of novel in-frame deletions; Figure S2: Distance monitors to track alteration of the iSH2 domain; Figure S3: Free Energy Landscapes for each class of genomic variant. The data file cached_subset_alpha_carbon_positions. RData contains C^α^ atom coordinates from our simulations at 300 K, and cached_subset_alpha_carbon_positions_360 K. RData contains similar data for our simulations at 360 K.
