# Supplementary material for: Structural and Dynamic Analyses of Pathogenic Variants in PIK3R1 Reveal a Shared Mechanism Associated among Cancer, Undergrowth, and Overgrowth Syndromes
Source: Life (Basel). 2024 Feb 23;14(3):297. doi: 10.3390/life14030297 (PMC10971029; doi:10.3390/life14030297)
Supplement: Supplementary file 1 [file life-14-00297-s001.zip › PIK3R1 cohort molecular modeling - SI.pdf]

Structural and Dynamic Analyses of Pathogenic Variants in *PIK3R1* Reveal a Shared Mechanism Associated among Cancer, Undergrowth, and Overgrowth Syndromes

Authors: Nikita R. Dsouza[1], Catherine E. Cottrell[2,3], Olivia M.T. Davies[4], Megha Tollefson[5], Ilona Frieden[6], Donald Basel[7], Raul Urrutia[1,8,9], Beth A. Drolet[10], Michael T. Zimmermann[1,9,11,*]

SUPPLEMENTAL DATA

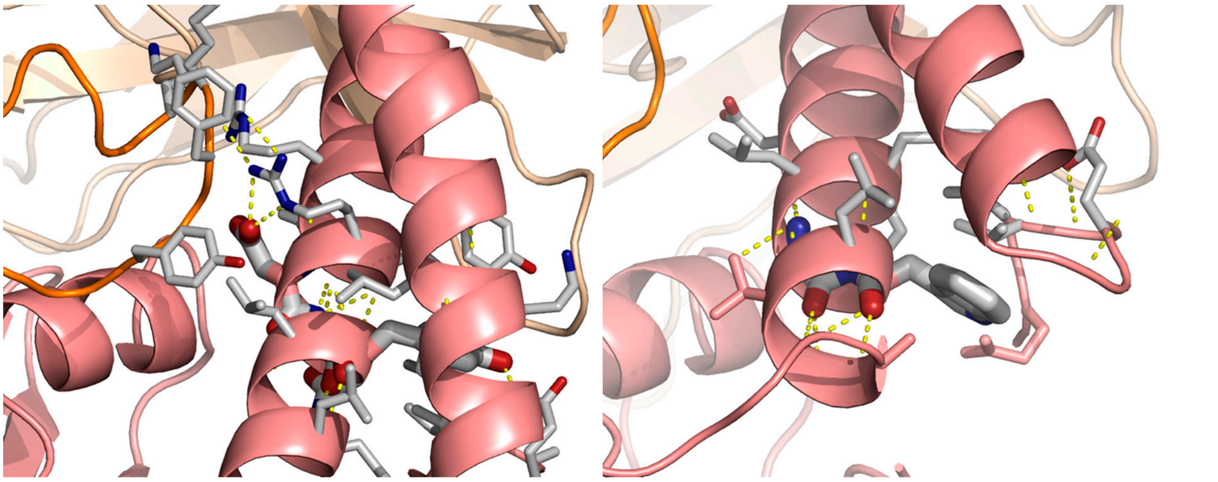

Figure S1: WT 3D context of novel in-frame deletions. A) The three deleted residues are shown in thicker sticks and surrounding residues in thin sticks. D578 and R577 make specific interactions with the activation loop. Additionally, Y580 is packed within the core of the PIK3R1 iSH2 domain. Loss of these three residues probably affects stability of the PIK3R1 head. Secondarily, it may affect the stability of the "open" conformation of the activation loop. **B)** The rest of the deleted residues make contacts within the receptor and their loss may destabilize the receptor. W582 is "the next rung down" on the same helix as Y580. It also makes many contacts and appears to be critical for the internal packing of the iSH2 domain.

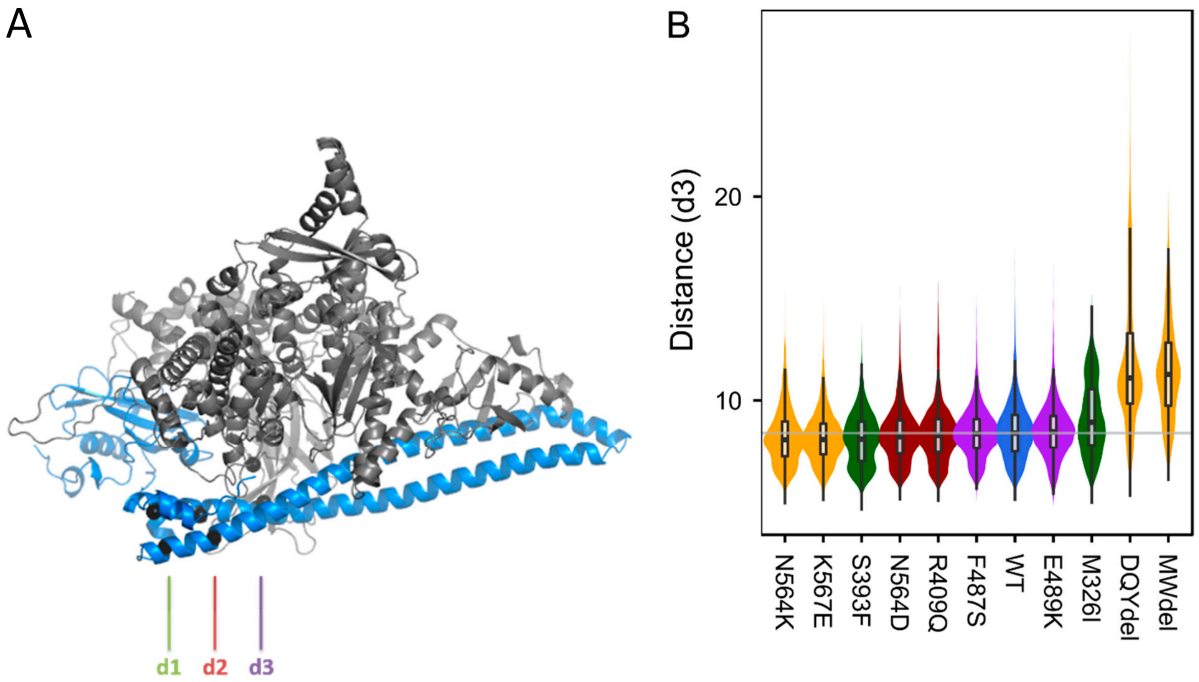

Figure S2: Distance monitors to track alteration of the iSH2 domain. A) Our model of the protein complex is shown with PIK3CA colored gray and PIK3R1 colored blue. Three pairs of residues within the iSH2 domain are shown and labeled as “d1” (PIK3R1-V445 to PIK3R1-L584), “d2” (PIK3R1-Y452 to PIK3R1-R577), and “d3” (PIK3R1-K567 to PIK3CA-E453). These three pairs were chosen because they are at the end of the coiled-coil motif and nearby the two in-frame INDELs (assuming both are normally spliced; MWdel may exon skip) that we have observed in association with the vascular malformation/overgrowth phenotype. **B)** The first distance pair is not affected significantly by any variants other than the two INDELs. Both INDELs are associated with a significantly greater separation of the two helices. This is indicative of local unfolding, even though it is the connecting loops that are shortened. Similar but attenuated differences are observed for the other distances monitored.

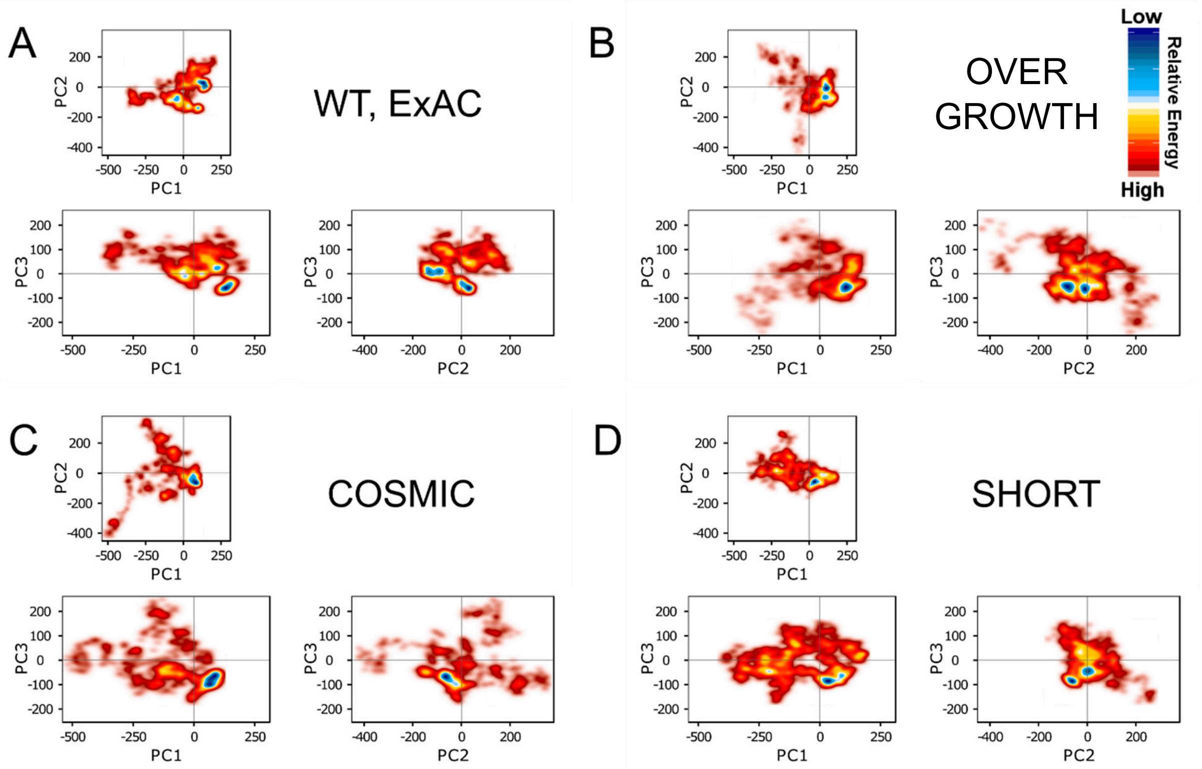

Figure S3: Free Energy Landscapes for each class of genomic variant. FEL diagrams are shown as in Figure 3. PCs are calculated across the entire dataset and each simulation projected onto them. Thus, for example, PC1 is the same in all panels. Specifically, we grouped variants as observed within **A)** ExAC only, **B)** overgrowth-associated pathogenic variants, **C)** somatically in cancer, and **D)** in association with the undergrowth syndrome, SHORT.
